# Common mistakes in biostatistics

**DOI:** 10.1093/ckj/sfae197

**Published:** 2024-06-26

**Authors:** Graziella D'Arrigo, Samar Abd El Hafeez, Sabrina Mezzatesta, Domenico Abelardo, Fabio Pasquale Provenzano, Antonio Vilasi, Claudia Torino, Giovanni Tripepi

**Affiliations:** CNR-IFC, Institute of Clinical Physiology of Reggio Calabria, Italy; Epidemiology Department, High Institute of Public Health, Alexandria University, Alexandria, Egypt; CNR-IFC, Institute of Clinical Physiology of Reggio Calabria, Italy; Department of Medical and Surgical Sciences, Magna Grecia University, of Catanzaro and Regional Epilepsy Center, Great Metropolitan BMM Hospital of Reggio Calabria, Italy; CNR-IFC, Institute of Clinical Physiology of Reggio Calabria, Italy; CNR-IFC, Institute of Clinical Physiology of Reggio Calabria, Italy; CNR-IFC, Institute of Clinical Physiology of Reggio Calabria, Italy; CNR-IFC, Institute of Clinical Physiology of Reggio Calabria, Italy

**Keywords:** methodological errors, mistakes in biostatistics, mistakes in clinical epidemiology

## Abstract

Biostatistics plays a pivotal role in developing, interpreting and drawing conclusions from clinical, biological and epidemiological data. However, the improper application of statistical methods can lead to erroneous conclusions and misinterpretations. This paper provides a comprehensive examination of the most frequent mistakes encountered in the biostatistical analysis process. We identified and elucidated 10 common errors in biostatistical analysis. These include using the wrong metric to describe data, misinterpreting *P*-values, misinterpreting the 95% confidence interval, misinterpreting the hazard ratio as an index of prognostic accuracy, ignoring the sample size calculation, misinterpreting analysis by strata in randomized clinical trials, confusing correlation and causation, misunderstanding confounders and mediators, inadequately codifying variables during the data collection, and bias arising when group membership is attributed on the basis of future exposure in retrospective studies. We discuss the implications of these errors and propose some practical strategies to mitigate their impact. By raising awareness of these pitfalls, this paper aims to enhance the rigor and reproducibility of biostatistical analyses, thereby fostering more robust and reliable biomedical research findings.

## INTRODUCTION

Biostatistics plays a crucial role in clinical and epidemiological research and provides the framework for analysing and interpreting data to gain knowledge in clinical and epidemiological science and patient care. However, common mistakes in biostatistics and the data analysis process can jeopardize the validity and reliability of study findings, potentially leading to erroneous conclusions and misguided clinical practices. Understanding these pitfalls is of paramount importance for clinicians and researchers alike to ensure robust study design, accurate analysis and meaningful interpretation of results.

In this paper, we highlight some of the common mistakes and misinterpretation encountered in biostatistics within the realm of clinical research, shedding light on areas where vigilance and attention to detail are critical. By recognizing these pitfalls and employing appropriate statistical methods, researchers can enhance the quality and integrity of their studies, ultimately contributing to evidence-based healthcare decision-making.

## OVERALL PROBLEMS THAT SHOULD BE AVOIDED

### Using the wrong metric to describe data

The first preliminary step in the statistical analysis process is to examine the data distribution. To avoid misuse of some summary measures (such as mean and median), the assessment of data distribution of a continuous variable can be performed by either visual (graphical) inspection using histograms, dots plots, or box and whisker plots, or [1] appropriate statistical tools (e.g. Shapiro–Wilk test). Furthermore, looking at the data distribution is important for data quality control, i.e. to identify wrong data due to typing or measurement errors, or the use of different units of measure for the same biomarker in the database (this typically occurs in multicentric studies). If data have outliers or the distribution is skewed, using the mean as a measure of location and standard deviation as an index of variability does not accurately summarize the data. This is because the mean and the standard deviation are measures affected by outliers. Compared with the mean, the median is much more robust against scattering. When the distribution of a given variable is skewed, the median and the interquartile range are the appropriate measures to summarize the data. However, if the distribution is approximately normal, the mean and the standard deviation are adequate measures of data summary. Choosing a wrong summary measure (for example, the mean instead of the median when the distribution is skewed) leads to choosing the incorrect statistical test for data comparison (e.g. *t*-test instead of Mann–Whitney test), thus providing misleading results.

### Misinterpreting *P*-values

In a clinical trial comparing two treatment arms for a given endpoint, the *P*-value (or type I error) is the probability of observing a between-groups difference as extreme or more extreme as what we observed, assuming that the null hypothesis is true [2]. Therefore, a *P*-value of .05 indicates that if the null hypothesis is true, there would be a 5% chance of observing the data or more extreme results purely due to random chance. It is important to note that the *P*-value does not provide information about the probability of the null hypothesis itself [3] and does not necessarily imply practical or clinical importance of the observed effect. Instead, the statistical significance merely suggests that the observed effect is unlikely to occur by chance alone. Thus, the significance of the effect must be interpreted in the context of its magnitude (effect size) and relevance to the research question [4]. A *P*-value above .05 may result from various factors, including a small effect size, a large amount of data variability or a small sample size. A *P*-value above .05 does not conclusively demonstrate the absence of a relationship or effect. Instead, it suggests that the evidence against the null hypothesis is not strong enough to reject it at the chosen significance level. Researchers should always examine the point estimate [e.g. mean difference, relative risk (RR), odds ratio, etc.] and its 95% confidence interval (CI) to assess the magnitude and precision of the observed effect.

### Misinterpreting the 95% confidence interval

In the context of a randomized clinical trial, the 95% CI is a measure of uncertainty of the effect of a given drug as assessed by risk ratio, hazard ratio, odds ratio or mean difference. Proper interpretation of the 95% CI involves understanding that if multiple samples were drawn from the same population and a 95% CI calculated for each sample, we would expect the population parameter to be found within 95% of these CIs [5]. Thus, the 95% CI does not imply that there is a 95% chance that the true population parameter falls within the interval [6]. The width of the 95% CI is influenced by various factors such as the sample size and data variability. A wider 95% CI may result from a smaller sample size or greater data variability. Conversely, a narrower 95% CI suggests more precision in estimating the population parameter and may result from a larger sample size or lower data variability. While a 95% CI that does not include a specific value (e.g. 0—for mean difference, or 1—for risk ratio, odds ratio and hazard ratio) indicates statistical significance, it does not automatically imply practical importance. Researchers should consider both statistical significance and the magnitude of effect when interpreting the 95% CI [7]. It is important to note that CIs and hypothesis testing serve different purposes in statistical inference. Hypothesis testing evaluates the evidence against a specific null hypothesis, while 95% CIs provide an interval estimate of a population parameter [8]. CIs rely on certain assumptions which interested readers can find elsewhere [9].

### Misinterpreting the hazard ratio as an index of prognostic accuracy

**Figure 1:**
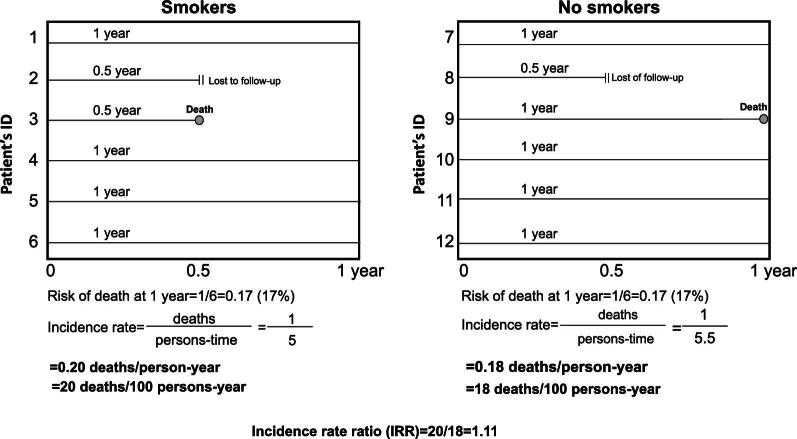
Description of the incidence rate (and IRR) calculation in two hypothetical groups of patients (see text for more detail).

The hazard ratio is conceptually like the incidence rate ratio (IRR) [10]. Consider an observational study examining the effect of smoking on 12-month mortality where there are six smokers and six non-smokers (Fig. 1). Over the study duration (12 months), among smokers, one patient is lost to follow-up after 6 months (0.5 year), one patient dies after 6 months (0.5 year) and the remaining four patients complete the 1-year follow-up. Among non-smokers, one patient is lost to follow-up after 6 months (0.5 year), one patient dies after 12 months (1 year) and the remaining four patients complete the 1-year follow-up (Fig. 1). To assess the risk of death, we calculate the probability of mortality in smokers (1/6 = 0.17, or 17%) and non-smokers (1/6 = 0.17, or 17%). Thus, the 1-year mortality is the similar in both groups and the risk ratio (an RR) is 1. The risk ratio ignores the observation that the death case occurs first in smokers (after 6 months) than in non-smokers (after 12 months). To consider the time to event, we calculate the total person-time (i.e. the sum of the times of observation of patients) in each group. The total person time is 5 years in smokers (0.5 + 0.5 + 1 + 1 + 1 + 1 = 5) and 5.5 in non-smokers (0.5 + 1 + 1 + 1 + 1 + 1 = 5.5). The incidence rate for smokers is 1/5 = 0.20 deaths/person-year (i.e. 20 deaths per 100 person-years), and the incidence rate among non-smokers is 1/5.5 = 0.18 deaths per person-year (i.e. 18 deaths per 100 person-years). The IRR is calculated by dividing the incidence rates of death in smokers and non-smokers (20/18 = 1.11). An IRR of 1.11 means that the probability of death occurring first is 11% higher in smokers than in non-smokers. Given the similarity between the two measures, the hazard ratio can be roughly interpreted as an IRR. Hazard ratios differ from RRs in that RRs are cumulative over an entire study, using a defined endpoint, while hazard ratios represent instantaneous risk over the study time, or some subset thereof. As with all relative measures of effect, the hazard ratio is an index of paramount importance in etiological research but *per se* does not provide information on the prognostic accuracy of a given exposure. Interested readers can find elsewhere a detailed review of statistical methods to be applied in prognostic research [11].

## ISSUES THAT SHOULD BE AVOIDED IN RANDOMIZED CLINICAL TRIALS

### Ignoring the sample size calculation

**Figure 2:**
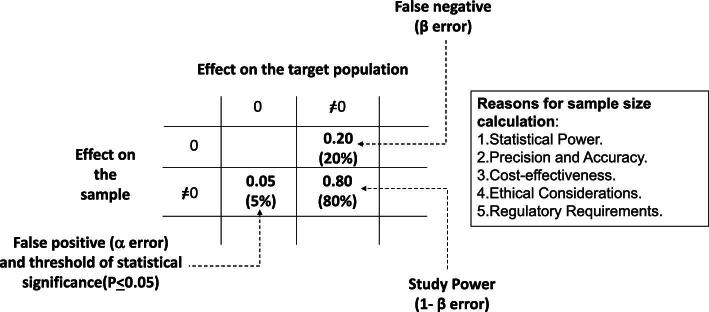
Explanation of the basic concepts underlying the sample size calculation (see text for more detail). The main reasons for the sample size calculation are also listed.

The basic concepts underlying the sample size calculation are explained graphically in Fig. 2. A researcher testing the efficacy of a certain drug on a specific outcome, can make two mistakes: (i) find a difference in the sample when there is not in the target population (i.e. to incur in a false positive or alpha error); and (ii) do not find a difference in the sample when there is in the target population (i.e. to incur in a false negative or beta error). The alpha and beta errors are set to 0.05 (5%) and to 0.20 (20%), respectively (Fig. 2). The complement to 1.0 of the beta error is the study power that must be at least 80% (Fig. 2). An 80% power means that if there is a difference (or an effect) between the arms being studied in the target population, the study sample has an 80% chance of finding it with an alpha error ≤0.05. Ignoring the sample size calculation [12] in a clinical trial can have significant ethical and scientific consequences. Randomized controlled trials (RCTs) must adhere to ethical guidelines to ensure the safety of participants. Ignoring sample size calculations can lead to enrolling too few participants, which may increase the risk to participants if the trial is underpowered to detect important effects or adverse events. This could lead to ethical concerns regarding the risk–benefit ratio for participants. Sample size calculations are crucial for ensuring that a study has sufficient statistical power to detect meaningful differences or effects. Ignoring these calculations can result in underpowered studies, where the sample size is too small to detect a clinically relevant effect even if it exists. As a result, the study may fail to provide reliable conclusions, leading to wasted resources and potentially misleading findings. Inadequate sample sizes increase the likelihood of false-positive or -negative results. A false-positive result occurs when the study erroneously concludes that a treatment is effective when it is not, leading to unnecessary adoption of ineffective treatments or interventions. Conversely, a false-negative result occurs when the study fails to detect a true effect, potentially denying patients access to beneficial treatments. Conducting clinical trials requires substantial resources, including time, money and effort, from researchers, participants and funding agencies. Ignoring sample size calculations can result in inefficient resource allocation, as studies may be underpowered to yield meaningful results. This wastes valuable resources that could be better utilized in well-designed studies. Failure to properly design a clinical trial, including determining an appropriate sample size, can damage the credibility of the study and the researchers involved. It may lead to skepticism from the scientific community, regulatory authorities and the public regarding the validity and reliability of the study findings. Regulatory bodies, such as the Food and Drug Administration in the USA, require justification for the chosen sample size in clinical trial protocols. Ignoring sample size calculations may result in regulatory non-compliance and delays in the approval process. When designing a clinical trial, it is also crucial to avoid including more patients than necessary for ethical considerations (enrolling more participants than needed exposes additional individuals to potential risks without additional benefit), resource allocation (over-enrollment can strain financial and logistical resources, diverting them from other potentially important research projects), statistical and time efficiency (over-enrolling can prolong the duration of the trial, delaying the availability of potentially beneficial treatments to the wider population). In summary, ignoring sample size calculations in a clinical trial can have serious consequences, including ethical concerns, unreliable results, wasted resources, and loss of credibility and efficiency. It is essential for researchers to carefully plan and justify sample sizes to ensure that their studies are adequately powered to yield meaningful and reliable conclusions.

### Misinterpreting analysis by strata in randomized controlled trials

In RCTs, stratified analysis aims to examine the effects of the intervention within subgroups defined by certain characteristics (e.g. males/females; age below/above 65 years, etc.) [13]. In an RCT with significant results, subgroup analyses can aid in pinpointing which patients experience greater benefit, those with lesser benefit or those who derive no benefit at all. Conversely, in an RCT with not-significant results, subgroup analyses can reveal specific patient groups where the experimental treatment shows effectiveness, thus providing the basis for a future, specifically designed, trial. By analysing treatment effects within different strata (subgroups), researchers can identify factors that modify the treatment effect. Thus, stratified analyses may reveal effect modification, where the magnitude or even direction of treatment effects vary across strata. While this can provide valuable insights into treatment heterogeneity, misinterpretation occurs when effect modification is mistaken for subgroup differences in treatment efficacy. A common mistake is to look at the statistical significance of the effect of the allocation arm on a given endpoint in a specific subgroups (e.g. in males versus females) rather than at the *P* for effect modification comparing the effects of treatment between males and females. This is because the statistical significance (and the 95% CI) of the effect in each subgroup crucially depends on the sample size of that subgroup. For example, if in an RCT the number of females is much smaller than that of males, the consequence is that the effect of a given drug does not achieve the statistical significance in females due to the low sample size. Furthermore, the randomization is expected to provide balanced treatment groups for measured and unmeasured factors in the overall study sample and we cannot be sure that this is also true between treated and untreated patients in males and females. Instead, focus should be on the consistency of treatment effects across strata and whether any observed differences are clinically meaningful. Finally, conducting multiple hypothesis tests within strata increases the risk of spurious findings due to multiple comparisons. Adjustments for multiplicity should be considered to control the overall type I error rate. Failure to account for multiplicity can lead to false-positive results and erroneous conclusions about treatment effects within strata. To mitigate misinterpretation of analysis by strata in RCTs, clear pre-specification of stratification factors and analysis plans, transparent reporting of methods and results, cautious interpretation of subgroup findings, and consideration of the overall trial design and potential biases are essential. Collaboration between statisticians, clinicians and researchers can help ensure appropriate analysis and interpretation of trial results.

## OVERALL ISSUES THAT ARISE IN OBSERVATIONAL RESEARCH IRRESPECTIVE OF THE DATA SOURCE

### Confusing correlation and causation

Correlation simply describes the association between types of variables that vary together. However, this association does not necessarily imply direct or indirect causal link [14]. On the other hand, causation means that changes in one variable (the determinant or independent variable) bring about changes in the other (the resultant or dependent variable), independently of potential confounders [15]. For example, cigarettes smoking correlates with the lung cancer risk, and it is causally implicated in the development of the disease. However, it is also true that cigarette smoking correlates with alcohol intake, but smoking *per se* does not cause alcoholism. Thus, establishing causation requires more than just observing a correlation between two variables. It often involves experimental studies, such as RCTs, to determine whether changes in one variable directly lead to changes in another, or (when RCTs are not feasible or are unethical) observational studies that consistently indicate that a given exposure–outcome correlation is independent of potential confounders and that such a causal relationship has a biological plausibility [15].

### Misunderstanding confounders and mediators

Confounding occurs in aetiological studies when a researcher aims at assessing the effect of an exposure on a given outcome, but inadvertently measures the effect of a third variable, i.e. the confounding variable (or ‘confounder’). A confounder is a variable associated with both the exposure and the outcome, but not in the same causal pathway. In medical studies, where the aim is often to explore the causes of a disease, thus establishing causal relationships, confounding is considered an undesirable phenomenon that needs to be ruled out [16]; for this reason, methods to control for confounding during the design of the study (i.e. by matching or restriction) or during the analysis phase (i.e. by stratification or adjusted analyses) are applied [17–19]. When a third variable is in the causal pathway between exposure and outcome, it is defined as ‘mediator’. In contrary to a confounder, a mediator explains the relation between a risk factor and the outcome variable, i.e. ‘how’ or ‘why’ one risk factor causes an outcome variable [19, 20]. Thus, a mediation analysis aims to understand the mechanism or process through which one variable influences another. To establish whether one variable is a confounder or a mediator is not always easy, but it depends on the knowledge of the involved pathogenetic mechanisms.

## ISSUES THAT SHOULD BE AVOIDED IN COHORT STUDIES

### Inadequately codifying variables during the data collection

Data collection is the process of gathering information from individuals as well as from medical and electronic records. Codifying variables is the process of assigning numerical codes or labels to different categories or values of variables that are collected to address the study aims. This codification helps in organizing data in a way that allows/facilitates statistical analysis. In a prospective cohort study, by assigning codes to variables, researchers transform qualitative data into a format that is more easily quantifiable for statistical analysis. If variables are not adequately codified, several problems can arise. It is generally recommended to avoid using string, and variables should always be recoded into numerical terms. For example, the qualitative variable ‘smoking’ can be recoded into a binary indicator variable (0, 1), where 1 represents smokers and 0 represents non-smokers. Ordinal variables, such as New York Heart Association (NYHA) classification for grading heart failure (no limitations in physical activity, mild limitations, moderate limitations and symptoms occur at rest) should be reported as 0, 1, 2 and 3. A clear legend explaining the meaning of recodification should be provided together with the database. If variables are not properly codified, researchers may need to spend more time and resources cleaning and preparing the data for statistical data analysis, delaying the research process and potentially increasing the probability of mistake. Missing data should be identified by an appropriate code or leaving empty the corresponding cells in the database. Attention should be paid to date format in survival analyses, and to the use of commas and points (depending on the country in which the data collection occurs) to indicate decimal numbers and thousands. Symbols such as ‘<’ or ‘>’ for a specific biomarker should be avoided and replaced by the smallest and highest measurable value of that biomarker being investigated. However, it is important noting that in a retrospective cohort study—unlike a prospective one—not all variables are measured the same way according to a predefined protocol, because data are collected for various clinical or epidemiological purposes. This implies that it is not possible to ‘codify’ all variables ahead of data collection, a circumstance leading to specific data issues. For example, kidney function impairment may not be properly coded, but only be evident when looking at laboratory test results; laboratory tests and other investigations are not carried out on everyone, and typically arise because of clinical suspicion and are done in the sicker population. This leads to selection bias and confounding. A framework for standardizing the reporting definitions of exposure and outcome in observational studies on kidney disease, utilizing routinely collected data, is described in detail elsewhere [20].

## BIAS ARISING WHEN GROUP MEMBERSHIP IS ATTRIBUTED TO THE BASIS OF FUTURE EXPOSURE IN RETROSPECTIVE STUDIES

Researchers are increasingly turning to routinely collected data from large databases to assess the effectiveness of specific interventions on a certain endpoint such as progression of chronic kidney disease [21]. A fundamental principle of these studies is that group membership (such as being exposed or unexposed to a given treatment) is determined by current exposure information rather than by future data. However, pharmacoepidemiologic studies using existing databases often violate this principle and failure to appropriately characterize medication use can induce both immortal time and selection bias.

### Introducing immortal time bias in retrospective cohort studies

**Figure 3:**
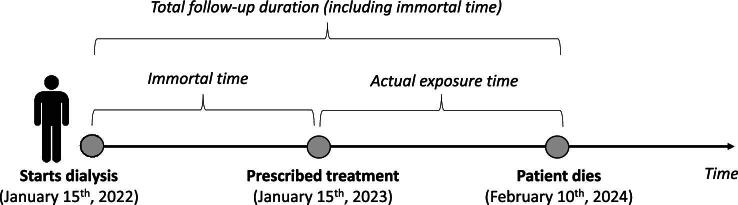
Graphical description of the immortal time bias (see text for more detail).

Immortal time bias occurs in retrospective, observational, cohort studies when the follow-up time is incorrectly classified [22]. Immortal time refers to a period during which, based on the study design and predefined selection criteria, the study endpoint cannot occur. Immortal time commonly occurs when assessing an individual's exposure status involves a wait period (e.g. waiting for the administration of a given drug) during which follow-up time is accrued. This waiting period is deemed ‘immortal’ because patients in the exposed group have to survive until the treatment of interest is administered. Consequently, participants are ‘immortal’ as they must survive long enough to receive the exposure of interest. This bias distorts the association between the treatment and the clinical endpoint. For example, in a retrospective cohort study, if a patient starts dialysis on 15 January 2022, receives the treatment of interest on 15 January 2023 and experiences the event (death) on 10 February 2024, the time period between 15 January 2022 and 15 January 2023 is an immortal time because by definition the patients cannot die in this period (Fig. 3). Thus, the actual exposure period of the patient concerned starts on 15 January 2023, and not on 15 January 2022. Immortal time bias can lead to overestimation of the protective effect of the exposure being studied, and it can affect the validity of study findings. To mitigate this bias, researchers need to carefully define exposure groups and appropriately account for the timing of exposure and outcome events in the study design and analysis.

### Introducing selection bias in retrospective cohort studies

Drugs are typically administered daily for an extended period, rendering them subject to variation over time. Each day, individuals make a choice about whether to adhere to their medication regimen or not, leading to alternating periods of medication intake and non-intake. This dynamic presents a concern known as the healthy adherer/sick non-adherer bias, a form of selection bias that can distort study results. This bias arises in two primary ways. First, by examining only those who consistently use medication, the analysis is restricted to individuals who tolerate long-term therapy and likely adopt other health-promoting behaviours potentially linked to the outcome under investigation. Secondly, by focusing solely on individuals who never utilize medications (‘never-users’), comparisons are restricted to individuals who either never require treatment or have medical reasons preventing treatment, neither group being an ideal comparator population and potentially having different baseline risks. Furthermore, excluding individuals from baseline cohorts based on later (future) medication usage in the study introduces the potential for selection bias by excluding person-time at risk. These factors collectively contribute to biases in divergent directions, making it challenging to discern the combined effects’ magnitude and direction. Recently, Hernan *et al*. [23] proposed a comprehensive framework to mitigate bias arising when group membership is determined on future exposure in retrospective studies, i.e. by aligning (i) the timing of cohort eligibility, (ii) treatment allocation (exposure cohort) and (iii) time zero (the start of follow-up). Adhering to this framework and applying specific statistical tools [24] aids researchers in addressing immortal time bias and minimizing the risk of selection bias.

## CONCLUSIONS

Mastering biostatistics is imperative for ensuring the integrity and reliability of research findings in the field of life sciences. This paper has highlighted some of the most prevalent mistakes encountered in biostatistical analyses. By being mindful of these common pitfalls, researchers can enhance the validity of their studies and contribute to the advancement of evidence-based medicine. It is essential for researchers to continually educate themselves on proper statistical methodologies and seek guidance from experienced biostatisticians when conducting and interpreting analyses. Ultimately, by addressing these common mistakes, we can strengthen the rigor and credibility of biomedical research, leading to more accurate conclusions and improved patient outcomes.

## Data Availability

No new data were generated or analysed in support of this research.
